# Supplementary material for: Globalization and employment nexus: Moderating role of human capital
Source: PLoS One. 2022 Oct 21;17(10):e0276431. doi: 10.1371/journal.pone.0276431 (PMC9586370; doi:10.1371/journal.pone.0276431)
Supplement: S1 Appendix — (DOCX) [file pone.0276431.s001.docx]

**Appendix 1**

**List of Countries**

Armenia, Bangladesh, Brunei Darussalam, China, Hong Kong, India, Indonesia, Iran, Iraq, Japan, Jordan, Kuwait, Malaysia, Mongolia, Pakistan, Philippines, Qatar, Russian Federation, Saudi Arabia, Singapore, Sri Lanka, Syria, Thailand, Turkey, Vietnam, Yemen.
