# Supplementary material for: Globalization and employment nexus: Moderating role of human capital
Source: PLoS One. 2022 Oct 21;17(10):e0276431. doi: 10.1371/journal.pone.0276431 (PMC9586370; doi:10.1371/journal.pone.0276431)
Supplement: Attachment — Submitted filename: Plus one comments.docx [file pone.0276431.s002.docx]

**Manuscript Number: PONE-D-22-21757**

**Globalization and Employment Nexus: Moderating Role of Human Capital.**

The main aim of this paper is to investigate analyzes the moderating role of human capital in globalization employment nexus in 26 Asian countries. The topic is interesting, and it is a good idea to investigate this role.

The empirical literature has been presented in a systematic manner but needs moe up to date citations. The discussion of the results is fine too. However, the FE and RE results maybe compared with the results of the system GMM if only the initial value of employment is included in these specifications. Also, the sentence structure must be improved and some grammatical errors removed in order to allow for the logical flow of ideas. I have pointed out some of these issues below. Furthermore, there is no policy implication and recommendations for further study.

It is a study that should be considered after **major corrections** have been made to address the following concerns:

1. The paper has not included a theatrical framework describing the link between globalization and employment and how the other variables might affect employment.
2. In the abstract, the authors stated that annual were collected from 1996 to 2019 using 12 specifications. How do we use specifications in the collection of data? Can they explain how a specification can be used to collect data? I suppose the authors meant the collected data were using in 12 specifications.
3. In lines 1 and 2 on page 8(introduction), Globalization is defined as the combination of capital flows, foreign direct investment, trade, technology, and labor mobility with the national economy. What does it mean by “with the economy? Or better still, they should include a citation.
4. In line 34 to 36 of page 9, the phase “The first of these takes into account the trade liberalization and the second foreign direct investment as the main indicators of economic globalization” should be rephrased to make more sense.
5. As far as the methodology is concerned, the authors have focused on the Hausman specification test and have not check the issues of heteroscedasticity, cross sectional dependence and serial correlation, if these issues are identified, the PCSE and GLS techniques are most appropriate techniques. Also, FE with Driscoll-Kraay standard errors can be used. These are completely absent.
6. In Lines 4 to 6 of the discussion paragraph on page 20, the statement “As far as indirect impact of globalization on employment creation through human capital via fixed effects model is in table 5 is concerned” is not understood. The authors have to fix this.
7. The statement ‘However, the impact of economic growth on negative on employment creation” in lines 22 to 23 of page 20 has to be revisited.
8. The policy implication and recommendations for further studies are absent.
