# Supplementary material for: Globalization and employment nexus: Moderating role of human capital
Source: PLoS One. 2022 Oct 21;17(10):e0276431. doi: 10.1371/journal.pone.0276431 (PMC9586370; doi:10.1371/journal.pone.0276431)
Supplement: Attachment — Submitted filename: PLOSONE_24 aug 2022.docx [file pone.0276431.s003.docx]

**Manuscript number: PONE-D-22-21757**

**Title: Globalization and Employment Nexus: Moderating Role of Human Capital**

General comment
This work is interesting work that needs **major revision** to be fit for publications in POLS ONE.

Recommendations: **Major revision**

1. Abstract

Abstract should start with problem statement and conclude with policy recommendations briefly. The organization of abstract is not satisfactory

2. Introduction

Introduction is not satisfactory authors should start with research problem along with providing detail rationale of the study. The novelty factor should be also highlighted properly.

3. Literature Review

The literature review section should discuss with theoretical model and discuss critically existing literature to highlight gap that this study aims to address. This section should explain novelty contribution of the study.

4. Methodology

The author should highlight source of equations, explain rationale why authors have used so many equations. Second, with test authors used to check multicollinearity problem. Also explain the rationale of variable used along with expected sign.

5. Results and Discussion

A real discussion of the findings is lacking. The discussion of empirical results indicates the lack of knowledge in your study area. Discussion and implications need to underline the contributions along with support from prior literature. Moreover, the diagnostics test analysis is missing here.

6. Conclusion

Conclusion part should highlight findings of this study that will lead to policy implications. Moreover, limitation of study and direction for future research should be properly highlighted.
