# Supplementary material for: Globalization and employment nexus: Moderating role of human capital
Source: PLoS One. 2022 Oct 21;17(10):e0276431. doi: 10.1371/journal.pone.0276431 (PMC9586370; doi:10.1371/journal.pone.0276431)
Supplement: Attachment — Submitted filename: Reply to reviewers comments PLOS One.docx [file pone.0276431.s004.docx]

Greetings, Editor

We appreciate your letting us submit a revision. Our anonymous expert's remarks were completely genuine, and we were happy to make the improvements they recommended.

We believe these changes in these revisions addressed everything necessary that was expected, but we want to reassure you that any new suggestions our experts may have would be welcomed.

The letter with our response and each item addressed, in turn, can be found below. In the manuscript text, we have color-coded both experts and provided numbers next to each of their comments to make things easier to comprehend.

Looking forward to starting revisions process

Regards,

Corresponding Author

| **Reviewer 1** | **Changings Recommended and reply** |
| --- | --- |
| Received Comments | 1. The paper has not included a theoretical framework describing the link between globalization and employment and how the other variables might affect employment. |
| Reply | 1. We appreciate our reviewer's thoughtful comments. You can track all of the red-highlighted points you made for us. As a result of answering point 1, we have provided the necessary theoretical framework. |
| Received Comments | 2. In the abstract, the authors stated that annual were collected from 1996 to 2019 using 12 specifications. How do we use specifications in the collection of data? Can they explain how a specification can be used to collect data? I suppose the authors meant the collected data were using in 12 specifications. |
| Reply | 2. We appreciate you making the remark and highlighting the problem you believe needs to be fixed. Correction has been made accordingly |
| Received Comments | 3. In lines 1 and 2 on page 8(introduction), Globalization is defined as the combination of capital flows, foreign direct investment, trade, technology, and labor mobility with the national economy. What does it mean by “with the economy? Or better still, they should include a citation. |
| Reply | 3. Thank you for your valuable point, this explanation was necessary. Correction has been made and citation has been included as you indicated |
| Received Comments | 4. In line 34 to 36 of page 9, the phase “The first of these takes into account the trade liberalization and the second foreign direct investment as the main indicators of economic globalization” should be rephrased to make more sense. |
| Reply | 4. thank you for identifying necessary corrections and need of sentences to be rephrased. Hence compliance has been made accordingly |
| Received Comments | 5. As far as the methodology is concerned, the authors have focused on the Hausman specification test and have not check the issues of heteroscedasticity, cross sectional dependence and serial correlation, if these issues are identified, the PCSE and GLS techniques are most appropriate techniques. Also, FE with Driscoll-Kraay standard errors can be used. These are completely absent. |
| Reply | 5. Thank you for indicting a better technique for this paper. Please follow our comments: Wald test for heteroscedasticity and Wooldridge test have been employed which pointed to presence of both problems. Therefore, generalized least squares (GLS) method has been applied for empirical analysis. |
| Received Comments | 6. In Lines 4 to 6 of the discussion paragraph on page 20, the statement “As far as indirect impact of globalization on employment creation through human capital via fixed effects model is in table 5 is concerned” is not understood. The authors have to fix this. |
| Reply | 6. Thank you for your emphasis, which may have reduced comprehension. Hence correction has been made accordingly. |
| Received Comments | 7. The statement ‘However, the impact of economic growth on negative on employment creation” in lines 22 to 23 of page 20 has to be revisited. |
| Reply | 7. The correction has been made considering your points in mind; we hope you will see them compellingly explaining the intended meaning. |
| Received Comments | 8. The policy implication and recommendations for further studies are absent. |
| Reply | 8. Thank you for highlighting shortcoming. As a result. We have provided Policy implication and recommendations for further studies considering recommendations in mind. |

We appreciate your thoughtful comments and Major and minor insights. We think this paper is a better version than the previous one, and we appreciate your guidance in making the manuscript better.

| **Reviewer 2** | **Changings Recommended and author reply** |
| --- | --- |
| Received Comments | 1. Abstract should start with problem statement and conclude with policy recommendations briefly. The organization of abstract is not satisfactory |
| Reply | 1. We appreciate your feedback and agree that your proposal is sound, thus we did what you suggested. |
| Received Comments | 2. Introduction is not satisfactory authors should start with research problem along with providing detail rationale of the study. The novelty factor should be also highlighted properly. |
| Reply | 2. Our entire team of coauthors concurred on this point, and it is appropriate to act as intended thus we followed your recommendation. We hope that these changes will be convincing enough for our expert requirements. |
| Received Comments | 3. The literature review section should discuss with theoretical model and discuss critically existing literature to highlight gap that this study aims to address. This section should explain novelty contribution of the study. |
| Reply | 3. This comment was taken into consideration and Corrections have been made as required. We will be waiting for your feedback. |
| Received Comments | 4. The author should highlight source of equations, explain rationale why authors have used so many equations. Second, with test authors used to check multicollinearity problem. Also explain the rationale of variable used along with expected sign. |
| Reply | 4. the recommendations have been followed; we will be looking forward for the feedback. |
| Received Comments | 5. A real discussion of the findings is lacking. The discussion of empirical results indicates the lack of knowledge in your study area. Discussion and implications need to underline the contributions along with support from prior literature. Moreover, the diagnostics test analysis is missing here. |
| Reply | 5. Thank you for your comments. We tried to overcome flaws you noticed and followed the recommendations have made. We expect that our efforts will be in accordance with what our reviewer anticipates. |
| Received Comments | 6. Conclusion part should highlight findings of this study that will lead to policy implications. Moreover, limitation of study and direction for future research should be properly highlighted. |
| Reply | 6. We see this point was equally important and highlighted by our other reviewer as well. We thank you for emphasizing for the need of policy implications. Findings of study, policy implications and direction of future research have been included. |

We value your insightful feedback and all of your insights. We believe that this work is an improvement over the previous one, and we value your suggestions for making the manuscript stronger.
