# Supplementary material for: Globalization and employment nexus: Moderating role of human capital
Source: PLoS One. 2022 Oct 21;17(10):e0276431. doi: 10.1371/journal.pone.0276431 (PMC9586370; doi:10.1371/journal.pone.0276431)
Supplement: Attachment — Submitted filename: Reply to reviewers comments PLOS One Round 2.docx [file pone.0276431.s005.docx]

Greetings, Editor

We appreciate your letting us submit a revision. Our anonymous expert's remarks were completely genuine, and we were happy to make the improvements they recommended.

We believe these changes in these revisions addressed everything necessary that was expected, but we want to reassure you that any new suggestions our experts may have would be welcomed.

The letter with our response and each item addressed, in turn, can be found below. In the manuscript text, we have color-coded both experts and provided numbers next to each of their comments to make things easier to comprehend.

Looking forward to starting revisions process

Regards,

Corresponding Author

| **Reviewer 2** | **Changings Recommended and reply** |
| --- | --- |
| Received Comments | 1. In the theoretical framework, the sentence “According to the Heckscher-Ohlin model, globalization increased employment in developing countries” should be According to the Heckscher-Ohlin model, globalization will increase employment in developing countries. |
| Reply | 1. We appreciate our reviewer's thoughtful comments. We change to “According to the Heckscher-Ohlin model, globalization will increase employment in developing countries” |
| Received Comments | 2. “Are” should be deleted from this sentence “However, the diagnostic tests are shown in tables 4 to 7 point out that the model suffers from heteroscedasticity and serial correlation problems”. |
| Reply | 2. We appreciate you making the remark and highlighting the problem you believe needs to be fixed. Correction has been made accordingly, we change to “However, the diagnostic tests shown in tables 4 to 7 point out that the model suffers from heteroscedasticity and serial correlation problems” |

We value your insightful feedback and all of your insights. We believe that this work is an improvement over the previous one, and we value your suggestions for making the manuscript stronger.
